# FUT11 expression in gastric cancer: its prognostic significance and role in immune regulation

**DOI:** 10.1007/s12672-024-01120-y

**Published:** 2024-06-28

**Authors:** Yanqing Huang, Xiaoying Yang, Mengda Wei, Xi Yang, Zhenmin Yuan, Junjie Huang, Junren Wei, Lei Tian

**Affiliations:** 1 The First Clinical Medical College, Guangxi Medical University, Nanning, 530021 Guangxi China; 2 Gastrointestine & Gland Surgery Division I, The First Affiliated Hospital of Guangxi Medical University, Nanning, 530021 Guangxi China

**Keywords:** FUT11, GC, Fucosylation, Immunology, Bioinformatics

## Abstract

**Background:**

Gastric cancer (GC) is a malignant digestive tract tumor with a high recurrence rate and poor prognosis. Fucosylation is important in tumor glycosylation, in which the key enzyme is fucosyltransferase (FUT). FUT11 is a member of the fucosyltransferase family and has been closely associated with the development of multiple cancers. However, the specific relationship between FUT11 and GC prognosis and its molecular mechanism has not been fully studied. This study explored FUT11 expression, clinical correlation, and its role in GC occurrence and development to deepen understanding of its function.

**Methods:**

FUT11 expression in 33 cancers was preliminarily analyzed using the Tumor Immunoassay Resource (TIMER2.0) database. FUT11 expression in GC was evaluated using The Cancer Genome Atlas stomach adenocarcinoma (TCGA-STAD) and Gene Expression Profiling Interactive Analysis (GEPIA2) data and verified using the Gene Expression Omnibus (GEO) GSE65801 dataset. Furthermore, we studied the survival prognosis of FUT11 in GC and analyzed its effect on the survival rate of patients with GC using the KM-plotter. We also performed COX regression analysis on TCGA GC clinical data and analyzed FUT11 expression in the pathway using the STRING and LinkedOmics databases. Moreover, the relationship between FUT11 and GC immune infiltration level was examined, and the Kaplan–Meier survival analysis diagram was constructed. The FUT11 genetic variation information was retrieved using cBioPortal, and its drug sensitivity was analyzed using CellMiner. Finally, differential FUT11 expression in GC tissues was verified using immunohistochemistry.

**Results:**

The data mining and analysis demonstrated that FUT11 expression was abnormally elevated in GC tissues and correlated with poor patient prognosis. The FUT11 expression level was an independent prognostic factor for GC. The difference in FUT11 expression level resulted in different degrees of immune cell infiltration in the patients with GC, which might regulate the tumor microenvironment. FUT11 affected GC development by participating in cancer pathways such as PI3K–AKT, neuroactive ligand–receptor, and MAPK. Immunohistochemical staining revealed that FUT11 was highly expressed in GC.

**Conclusions:**

This study revealed that FUT11 expression is significantly increased in GC tissues. This increase is associated with poor prognosis and might affect immune regulation. FUT11 might have immunological and targeted therapeutic value, providing a new approach to GC treatment.

**Supplementary Information:**

The online version contains supplementary material available at 10.1007/s12672-024-01120-y.

## Introduction

Gastric cancer (GC) is the fifth most common cancer worldwide and the third leading cause of cancer death [1]. The GC morbidity and mortality rates are among the top five in the world [2]. The current standard treatment for GC is radical tumor resection with perioperative chemotherapy, while the standard treatment for metastatic or unresectable GC includes chemotherapy regimens such as platinum-based drugs, docetaxel, paclitaxel, and irinotecan. The 5-year overall survival rate (OS) for patients with early-stage GC who undergo surgery is 90%. However, the lack of specific early clinical manifestations in patients with GC results in many patients only being diagnosed when the disease is relatively advanced and there is no longer an opportunity for radical surgical intervention. Advanced GC grows rapidly, has a high degree of malignancy, is difficult to treat, and has a poor prognosis.

GC occurrence and prognosis are closely related to abnormal gene expression in patients [3, 4]. However, the molecular mechanism of GC carcinogenesis remains unclear. Therefore, it is necessary to elucidate these mechanisms and search for molecular markers that can aid early diagnosis and prognosis assessment. Glycosylation is a well-regulated cellular and microenvironment-specific post-translational modification [5]. Abnormal protein glycosylation regulates the malignant cancer cell phenotype and is crucial in cancer cell interactions and tumor angiogenesis. Additionally, abnormal protein glycosylation is closely related to cancer cell immune evasion [6].

Fucosylation is key in tumor-associated abnormal glycosylation. Fucosyltransferase (FUT) is one of the most important enzymes that coordinates fucosylation. The abnormal protein modification by FUT is closely related to cancer occurrence and development. FUT is a key enzyme that catalyzes the transfer of l-fucose from the donor substrate β-l-fucose guanosine diphosphate to its respective substrate [7]. Glycosylation can be divided into core and terminal fucosylation according to the fucose location. Of the 13 identified FUTs, FUT1–11 undergo *N*-linked fucosylation in the Golgi apparatus [8], while *O*-linked fucosylation is catalyzed by *O*-FUTs in the endoplasmic reticulum. FUT8 is involved in terminal fucosylation, while the remaining FUT catalyzes core fucosylation [9]. Abnormal fucosylation is closely associated with various cancers. For example, FUT8 is essential for the increased synthesis of cancer-related *N*-glycan fucosylation, and high *N*-glycan fucosylation levels can be used as a biomarker to detect colon cancer onset [10, 11]. In breast cancer, inhibiting FUT8 expression affects cell migration by altering E-cadherin core fucosylation and affecting the downstream FAK–integrin pathway [12]. FUT11 has received much attention for its role in promoting hepatocellular carcinoma progression [13]. Another study reported that FUT11 was involved in pancreatic cancer development by stabilizing PDK1 [14]. PuZhang highlighted the poor prognosis associated with FUT11 in renal cell carcinoma [15]. These studies collectively emphasize the contribution of FUT11 to the origin and development of various cancers. However, the specific relationship between FUT11 and the prognosis of patients with GC, and its potential molecular mechanisms in GC development, remain unexplored.

In this study, we extensively analyzed FUT11 expression in GC using sequencing datasets. We explored abnormal FUT11 expression in GC tissues, its correlation with the clinical and pathological features of patients with GC, and its effects on the prognosis of such patients. We also investigated the co-expressed genes of FUT11 in-depth, carefully studying their potential molecular functions through Gene Ontology (GO) and Kyoto Encyclopedia of Genes and Genomes (KEGG) analyses. Furthermore, we examined the interaction between FUT11 and tumor-infiltrating immune cells. The results reveal the potential role and prognostic significance of FUT11 in tumor immunology, providing valuable insights into the underlying mechanisms of GC development.

## Materials and methods

### Expression analysis of FUT11 in GC

We analyzed FUT11 mRNA levels in GC using the Tumor Immunoassay Resource (TIMER2.0, https://cistrome.shinyapps.io/timer/), The Cancer Genome Atlas (TCGA, https://portal.gdc.cancer.gov/), and Gene Expression Profiling Interactive Analysis (GEPIA2, http://gepia.cancer-pku.cn/) databases [16]. Pan-cancer analysis using TIMER2.0 revealed that FUT11 was highly expressed in GC tissues compared to adjacent normal tissues. To validate this finding, we analyzed 408 GC and 211 normal gastric tissue samples using GEPIA2. Although the TIMER2.0 and GEPIA2 databases are based on TCGA data, they are online platforms with potentially different data filtering methods compared to the raw TCGA-STAD (stomach adenocarcinoma) data we downloaded. Subsequently, we conducted a cancer-specific differential expression analysis using the TCGA-STAD dataset, which included 413 pairs of GC tissues and adjacent tissues. As TCGA-STAD data primarily originates from individuals of American descent, while Gene Expression Omnibus (GEO) microarray data is predominantly from Chinese populations, we also analyzed a GEO dataset (GSE65801) that comprised 32 GC and 32 normal gastric tissue samples (https://www.ncbi.nlm.nih.gov/geo/) [17]. This analysis and the GEPIA2 results verified our findings.

### Survival data analysis of FUT11 gene expression in patients with GC

GEPIA2 is a web-based tool that aided the standardized analysis of RNA sequencing (RNA-seq) data of 9736 tumor samples and 8587 normal control samples in TCGA and Genotype-Tissue Expression (GTEx) datasets [18]. In the present study, we used GEPIA2 to determine the prognostic value of FUT11 expression in patients with GC. We divided the patients into high and low FUT11 expression groups according to the median FUT11 expression to explore the potential association between FUT11 expression level and the prognosis of patients with GC. Furthermore, we combined TCGA-STAD clinical data to analyze the influence of FUT11 expression on the adverse prognosis of patients with GC and constructed the dependence receiver operating characteristic (ROC) curve for 1, 2, and 3 years to more accurately reveal the role of FUT11 in GC occurrence and development.

### Clinical data analysis of FUT11 in patients with GC

The Kaplan–Meier plotter (KM-plotter) online database (http://kmplot.com/analysis/) is based on the GEO, European Genome-phenome Archive (EGA), and TCGA public gene chip databases, and RNA-seq was constructed from the data. KM-plotter was used to evaluate the effects of a single gene on the survival of patients with GC [19]. The database includes data on 1440 patients with GC. We explored the specific effects of FUT11 expression level on the survival rate of patients with GC using the probe ID238551_At in the KM-plotter database for detailed analysis, and the data were mainly from multiple datasets: GSE14210 (N = 145), GSE15459 (N = 200), GSE22377 (N = 43), GSE29272 (N = 268), GSE51105 (N = 94), and GSE62254 (N = 300).

Using the best cut-off values provided by the database, FUT11 expression levels were divided into high and low groups. Log-rank P-values and hazard ratios (HR) for 95% confidence intervals were calculated, and a three-line table of HR and P-values for Kaplan–Meier analysis was plotted based on different sets of clinical variables to present the results more intuitively. Additionally, more comprehensive information was obtained by collecting clinical data related to FUT11 expression in TCGA-STAD patients. After deleting missing data, we obtained valid data for 348 patients. Subsequently, univariate and multivariate COX regression analyses were conducted using the survminer, survival, and rms packages in R language to reveal the relationship between FUT11 expression and the clinical characteristics of patients with GC.

### Protein interaction network and hub gene analysis of FUT11

Possible protein interactions with FUT11 were collected and integrated using the STRING database (https://string-db.org/) [20]. First, the search module was used to search for individual protein names (“FUT11”) and organisms (“Homo sapiens”). Then, the following options were set: active interaction source (“Text mining and experimentation”), minimum interaction score required (“Low confidence (0.150)”), maximum number of interactions to display (“No more than 50 interactors” in the first option), and “default” for the other options.

The obtained relevant genes were used to analyze the protein–protein interaction (PPI) network, where a confidence score > 0.7 was set as a significance threshold. Finally, the relevant data were imported into Cytoscape (v3.9.1) for visualization and further analysis. Key modules were identified using the Cytoscape cytoHubba plugin, where the top 10 nodes in the cytoHubba maximum clique centrality (MCC) ranking served as hub genes. GO functional and KEGG enrichment analyses were conducted on the genes closely interacting with FUT11 obtained from STRING using the R clusterProfiler and org.Hs.eg.db packages. The cut-off threshold for the GO and KEGG pathway enrichment analyses was set to P < 0.01, and the results were displayed as a bubble plot through ggplot2.

The diagnostic value of the top 10 hub genes for GC was evaluated using the ROC curve. The data used to construct the ROC curve were from the mRNA expression of relevant genes in corresponding cancer tissues and normal tissues in the TCGA-STAD dataset. The ROC curve was calculated using the R package pROC (v1.17.0.1) and drawn using the package ggplot2. The area under the curve (AUC) was calculated, where an AUC closer to 1 indicated a better diagnosis. The accuracy was lower for AUC of 0.5–0.7, better for AUC of 0.7–0.9, and higher for AUC of ≥ 0.9.

### Gene co-expression analysis of FUT11 in GC

FUT11 gene expression or transcription in GC was analyzed using LinkedOmics [21] (http://www.linkedomics.org/login.php) and its related tools. After in-depth analysis, we obtained a total of 10,801 genes up-regulated in co-expression with FUT11 and 9424 genes down-regulated in co-expression with FUT11.These genes were visualized using heat maps generated using the top 50 genes with the most significant upregulated and downregulated expression. Subsequently, GO function and KEGG enrichment analyses of the LinkedOmics-screened genes that closely interacted with FUT11 were conducted using the R clusterProfiler and org.Hs.eg.db packages. The cut-off threshold for the GO and KEGG pathway enrichment analysis was set to P < 0.01, and the results were displayed as a bubble plot using ggplot2 to provide important clues to understand the molecular mechanism of FUT11 in GC.

### Immunofiltration analysis of FUT11 in GC

Tumor immune to assess resources (TIMER2.0) (https://timer.cistrome.org/) [16, 22, 23] including from the cancer genome atlas (TCGA) 10897 samples, to analyze genetic data of the table, And an estimated 33 cancer types infiltrate immune cells from tumors. The correlation between FUT11 expression and the immune infiltration levels in patients with GC was explored using the TIMER gene module. Furthermore, the correlation between FUT11 expression and immune infiltration in TCGA was analyzed using the immune gene module combined with various algorithms. Immune infiltration scores and lollipop plots for 24 immune cells were plotted using TCGA-STAD data and the single-sample gene set enrichment analysis (ssGSEA) algorithm. Finally, a forest map of Kaplan–Meier survival analysis in patients with GC with high FUT11 expression under different immune cell subsets was constructed using the KM-plotter database, strongly supporting GC immunotherapy.

### Genetic variation of FUT11 in GC

FUT11 genetic alteration information was retrieved using cBioPortal (https://www.cbioportal.org/) [24, 25] and included all TCGA pan-cancer studies. Genomic information on FUT11 alterations in cancer was explored using the Cancer Types Summary & Mutations and mRNA Expression & lncRNA Expression modules. Furthermore, the mutation module provided detailed information on the FUT11 mutation site to improve understanding of its potential role in GC development.

### Drug sensitivity analysis of FUT11

FUT11 expression and drug data were downloaded from CellMiner (https://discover.nci.nih.gov/cellminer/home.do) [26],  which includes 60 different cell lines and provides a one-stop resource for molecular and pharmacological data on the widely studied NCI-60 cancer cell panel. The correlation between FUT11 gene expression and drug median inhibitory concentrations (IC50) was analyzed using the R oncoPredict package. A correlation scatter plot was constructed for drug sensitivity analysis to enhance understanding of the mechanism of action of FUT11 in drug therapy.

### FUT11 expression in human GC tissue

Human GC tissues and adjacent tissue microarray chips (25 pairs of GC and adjacent tissues) were purchased from Suzhou CoWin Biotech Co., Ltd. The inclusion criteria were: all patients were diagnosed with GC by experienced pathologists and staged according to the eighth edition AJCC (American Joint Committee on Cancer) tumor-node-metastasis (TNM) staging system for GC, and had not received any treatment. The exclusion criteria were: incomplete clinical and pathological data.

The study received ethical approval from the First Affiliated Hospital of Guangxi Medical University Medical Ethics Committee (2023-E573-01). FUT11 immunohistochemical staining was performed using rabbit polyclonal anti-FUT11 antibody (catalog number 17175-1-AP, Sanying, Wuhan, China) at a 1:100 dilution ratio. The immunohistochemical results were analyzed using ImageJ, and the obtained average optical density (AOD) value (spectrophotometric value integrated OD [IOD]/area) was used for semi-quantitative visual analysis. Furthermore, the relationship between FUT11 expression and the clinicopathological features of patients with GC was analyzed using the χ^2^ test. As the specimens were all collected within 1 year, survival prognosis analysis could not be performed.

### Statistical analysis

The FUT11 gene expression pattern was determined by analyzing TCGA expression data. Survival curves with HR values, log-rank tests, or Cox regression analysis P-values were plotted using KM-plotter tools. The TIMER2.0 database results were evaluated using Spearman correlation analysis and a significance test after adjusting for tumor purity. The GEPIA2 database yielded HR results obtained through Spearman correlation analysis and corresponding P-values. All gene expression data were standardized by log2 conversion. All statistical analyses were performed using R software. The relationship between FUT11 expression and the clinicopathological features of patients with GC was analyzed using the χ^2^ and Wilcoxon rank sum tests. The bivariate correlation between the two variables was evaluated using the Spearman rank correlation coefficient. The difference was considered statistically significant at P < 0.05.

## Results

### Differential expression of FUT11 in GC

**Fig. 1:**
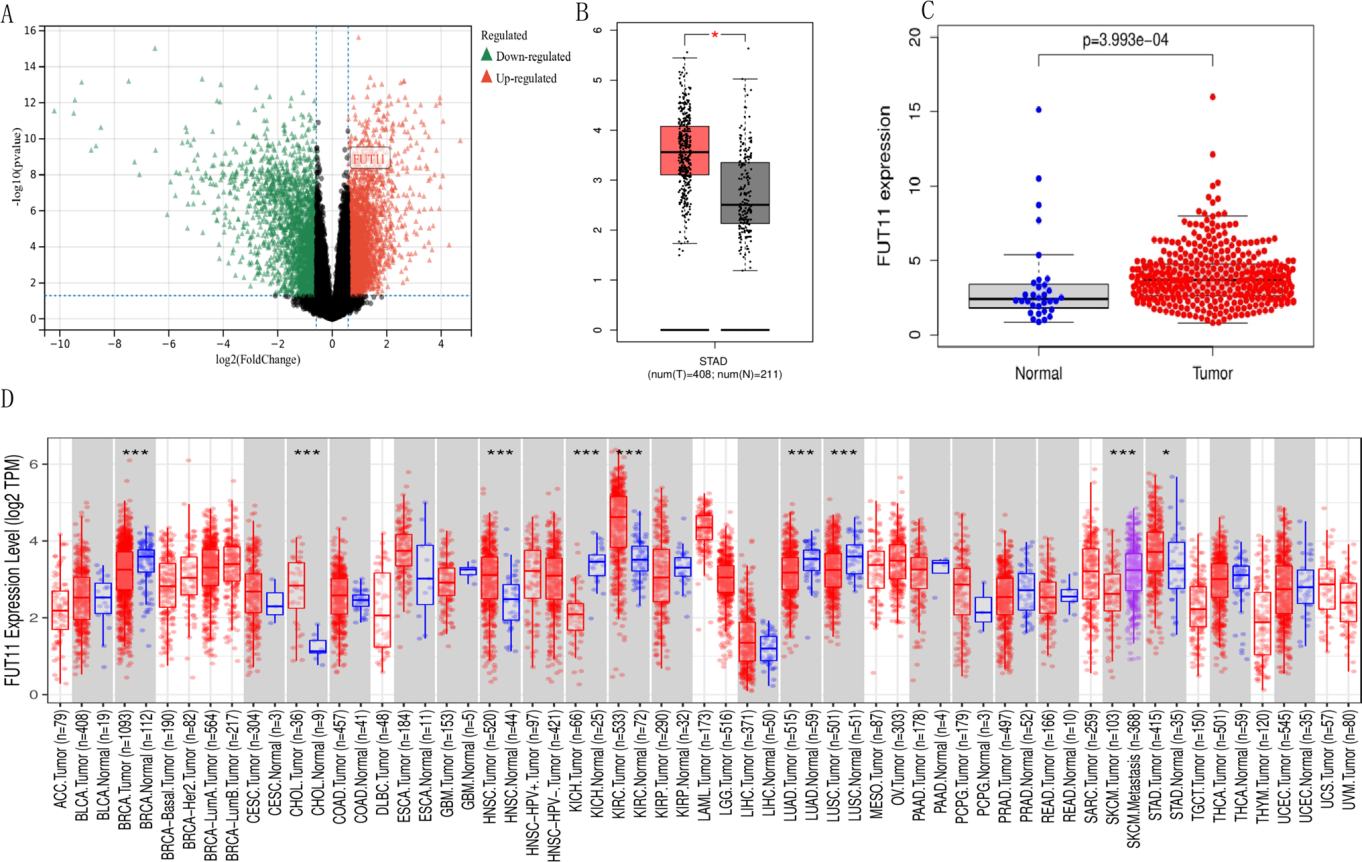
FUT11 is highly expressed in GC tissues. **A** Based on the GEO-GSE65801 dataset, differential expression of FUT11 in gastric cancer and adjacent tissues (P < 0.05); **B** Based on the GEPIA2 database, differential expression of FUT11 in gastric cancer and adjacent tissues (P < 0.05); **C** Differential expression of FUT11 in gastric cancer and adjacent tissues based on TCGA-STAD (P < 0.05); **D** The expression differences of FUT11 in cancer tissues and adjacent tissues of different cancer species in the TIMER2 database. (* P < 0.05, * * P < 001, * * * P < 0.001, * * * P < 0.0001)

A detailed study of the differences in FUT11 expression between normal and tumor tissues of 33 different cancers was conducted using the TIMER2.0 database to determine whether FUT11 is differentially expressed in GC. The analysis revealed that FUT11 expression in CHOL, HNSC, KIRC, and STAD was significantly higher than that in the normal control group. However, FUT11 expression in BRCA, KICH, LUAD, LUSC, and SKCM was significantly lower than that in normal control tissues (Fig. 1D). These results were validated, and the FUT11 expression pattern was confirmed using TCGA-STAD (413 patients with GC), GEPIA2 (408 GC tissues and 211 normal gastric tissues), and GSE65801 (32 GC tissues and 32 non-cancerous tissues), which confirmed the increased FUT11 expression in GC. The results were statistically significant (P < 0.05) (Fig. 1A–C) and indicated that FUT11 is upregulated in GC tissues.

### Survival analysis of FUT11 expression in patients with GC

**Fig. 2:**
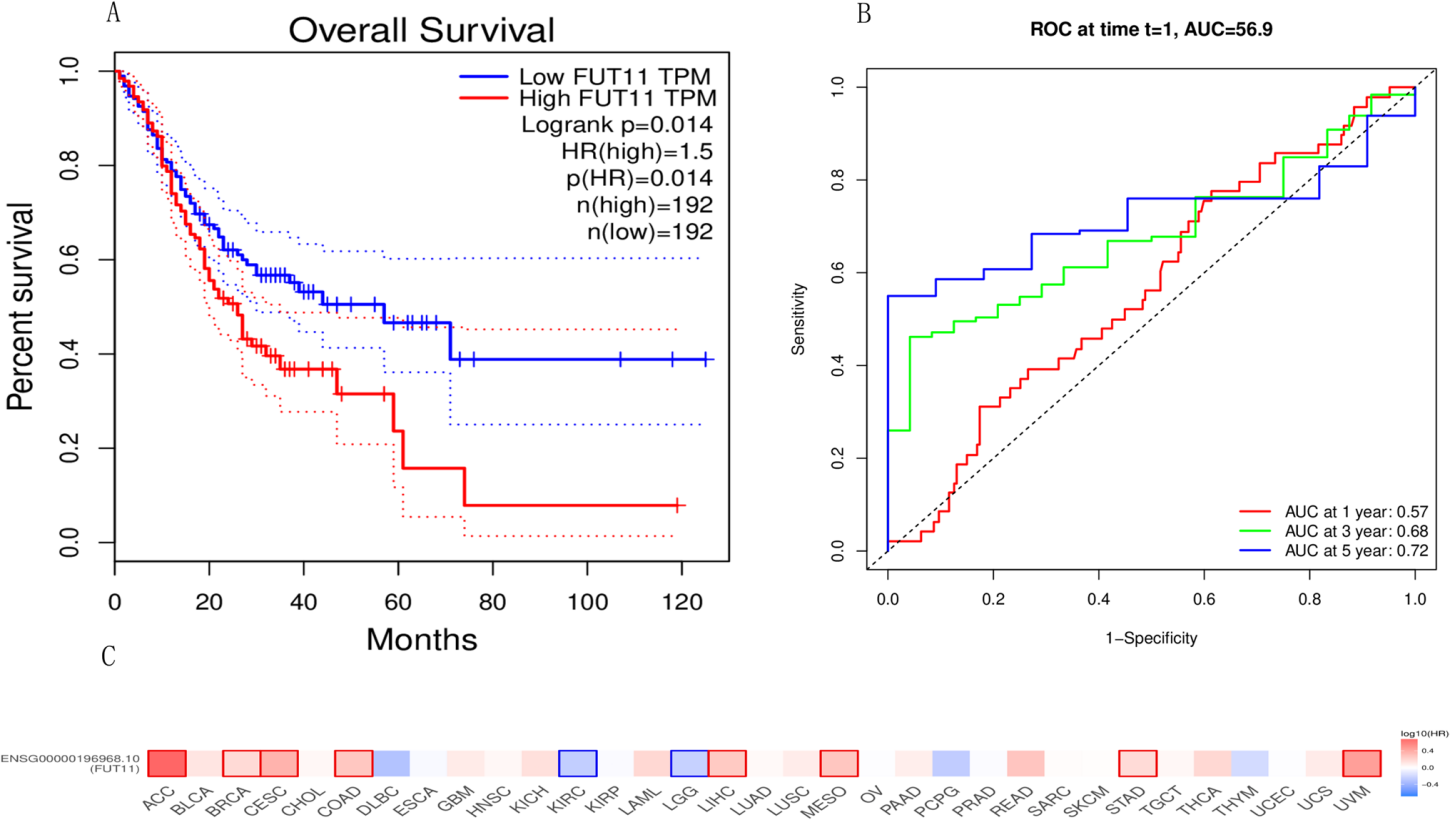
Survival analysis of high and low expression of FUT11 in GC patients. **A** Based on the GEPIA2 database, survival curves (OS) of GC patients with high and low expression of FUT11; **B** Time dependent ROC curves (1, 3, 5 years) of FUT11 in GC patients based on TCGA-STAD; **C** Based on the GEPIA2 database, heatmaps for survival analysis of high and low expression of FUT11 in different cancers

Based on the high FUT11 expression in GC tissues, we explored the effects of FUT11 on the survival of patients with GC. The association between FUT11 and the outcomes of patients with GC by conducting in-depth analysis using the GEPIA2 database. The results demonstrated that increased FUT11 expression was significantly associated with poor OS in ACC, BRCA, CESC, COAD, LIHC, MESO, STAD, and UVM (P < 0.05) (Fig. 2C). In GC, we observed a significant association with significantly reduced OS when FUT11 gene expression was higher than the median for the whole dataset (P < 0.05) (Fig. 2A). The FUT11 dynamics in the prognosis of patients with GC were assessed more fully using a time-dependent ROC analysis. The results demonstrated that the value of FUT11 in predicting patient prognosis increased over time, where the AUC values of the 1-, 3-, and 5-year time-dependent ROC curves were 0.57, 0.68, and 0.72, respectively (Fig. 2B). Analysis of the overall data demonstrated that high FUT11 expression was associated with poor prognosis in patients with GC, and the prognostic prediction value improved with survival time.

### Clinical correlation of FUT11 expression in patients with GC

We explored the correlation between FUT11 expression and the clinical features of prognosis in patients with GC. We divided the patients into high and low FUT11 expression groups according to the best cut-off value. The KM-plotter analysis demonstrated that increased FUT11 expression was significantly different between PF, OS, and PPS and was associated with poorer survival outcomes (Supplementary Fig. 1A–C), which was consistent with our previous analysis.

Additionally, we examined the relationship between FUT11 expression and tumor characteristics and prognosis. We determined that FUT11 expression was significantly correlated with T stage, N stage, and Lauren’s classification and was associated with poor PF and OS (Table 1). Further analysis demonstrated that FUT11 expression was more strongly correlated with N stage 1, 2, and 3, which corresponded to the degree of lymph node metastasis in patients with GC. This lymph node metastasis is the most common type of metastasis in patients with GC, and the overall HR of stage N was the highest, suggesting that FUT11 expression may affect the prognosis of patients with GC by affecting lymph node metastasis.

**Table 1:** The impact of FUT11 gene expression on clinical prognosis in gastric cancer under different clinical pathological factors

Clinicopathological characteristics	Overall survival (n = 631)			Progression free survival (n = 532)		
	N	Hazard ratio	P-value	N	Hazard ratio	P-value
Stage						
1	62	0.7 (0.23–2.15)	0.53	60	0.67 (0.22–2.05)	0.48
2	135	1.9 (1–3.6)	0.047	131	1.82 (0.99–3.36)	0.051
3	197	1.77 (1.22–2.58)	0.0025	186	1.88 (1.3–2.72)	7.00e−04
4	140	1.7 (1.14–2.54)	0.0081	141	1.64 (1.11–2.41)	0.011
Stage T						
2	241	1.6 (1.02–2.5)	0.037	239	1.61 (1.05–2.47)	0.029
3	204	1.62 (1.14–2.29)	0.0061	204	1.46 (1.04–2.04)	0.027
4	38	1.72 (0.73–4.04)	0.21	39	4.22 (1.84–9.71)	0.00025
Stage N						
0	74	0.62 (0.24–1.62)	0.33	72	1.53 (0.67–3.51)	0.31
1	225	2.13 (1.41–3.22)	0.00023	222	2.06 (1.4–3.04)	2.00e−04
2	121	2.23 (1.4–3.54)	0.00051	125	2.22 (1.43–3.44)	0.00027
3	76	1.91 (1.12–3.27)	0.016	76	2.08 (1.22–3.54)	0.0062
Stage M						
0	444	1.94 (1.46–2.56)	2.40e-06	443	1.95 (1.49–2.54)	5.60e−07
1	56	1.8 (0.97–3.31)	0.057	56	1.38 (0.74–2.57)	0.31
Lauren classification						
Intestinal	269	2.46 (1.69–3.57)	1.00e−06	263	2.34 (1.63–3.36)	2.00e−06
Diffuse	240	1.36 (0.95–1.93)	0.088	231	1.49 (1.05–2.11)	0.024
Mixed	29	0.23 (0.07–0.73)	0.0065	28	0.16 (0.05–0.57)	0.0013
Differentiation						
Poor	121	1.54 (0.93–2.54)	0.094	121	1.53 (0.94–2.47)	0.082
Moderate	67	2.1 (1.1–4.02)	0.021	67	1.92 (1.03–3.58)	0.038
Gender						
Female	187	1.42 (0.91–2.2)	0.12	179	1.46 (0.95–2.23)	0.079
Male	349	2.03 (1.49–2.75)	4.1e−06)	341	2.03 (1.52–2.72)	1.10e−06

The univariate COX regression analysis demonstrated that TNM stage and FUT11 expression level correlated with the prognosis of patients with GC. The multivariate COX regression analysis demonstrated that the FUT11 expression level was an independent risk factor for patients with GC and significantly affected their prognosis (Table 2). These results emphasize the important relationship between FUT11 and poor prognosis in patients with GC, establish FUT11 as a valuable predictor of GC prognosis, and provide a new perspective for assessing GC prognosis and formulating treatment strategies.

**Table 2:** COX univariate and multivariate analysis of FUT11 expression with age, gender, differentiation level, and tumor TNM staging in GC patients

Parameter	Univariate analysis			Multivariate analysis		
	HR	95%CI	P	HR	95%CI	P
Age	0.9933	0.9800–1.0069	0.3342	0.9927	0.9792–1.0064	0.2964
Gender	1.1474	0.8085–1.6282	0.4414	1.0882	0.7570–1.5644	0.6480
Grade	1.0578	0.7711–1.4511	0.7277	0.8759	0.6307–1.2166	0.4294
Pathological stage	1.4267	1.1794–1.7260	0.0003	1.1014	0.7572–1.6022	0.6133
T	1.5174	1.2194–1.8882	0.0002	1.4622	1.0955–1.9516	0.0099
N	1.0039	0.5537–1.8201	0.9899	0.5468	0.2637–1.1337	0.1046
M	1.2684	1.0932–1.4718	0.0017	1.1980	0.9716–1.4772	0.0909
FUT11	1.0348	1.0012–1.0696	0.0422	1.0517	1.0127–1.0921	0.0090

### FUT11 protein interaction network, functional enrichment, and the diagnostic value of hub genes in GC

**Fig. 3:**
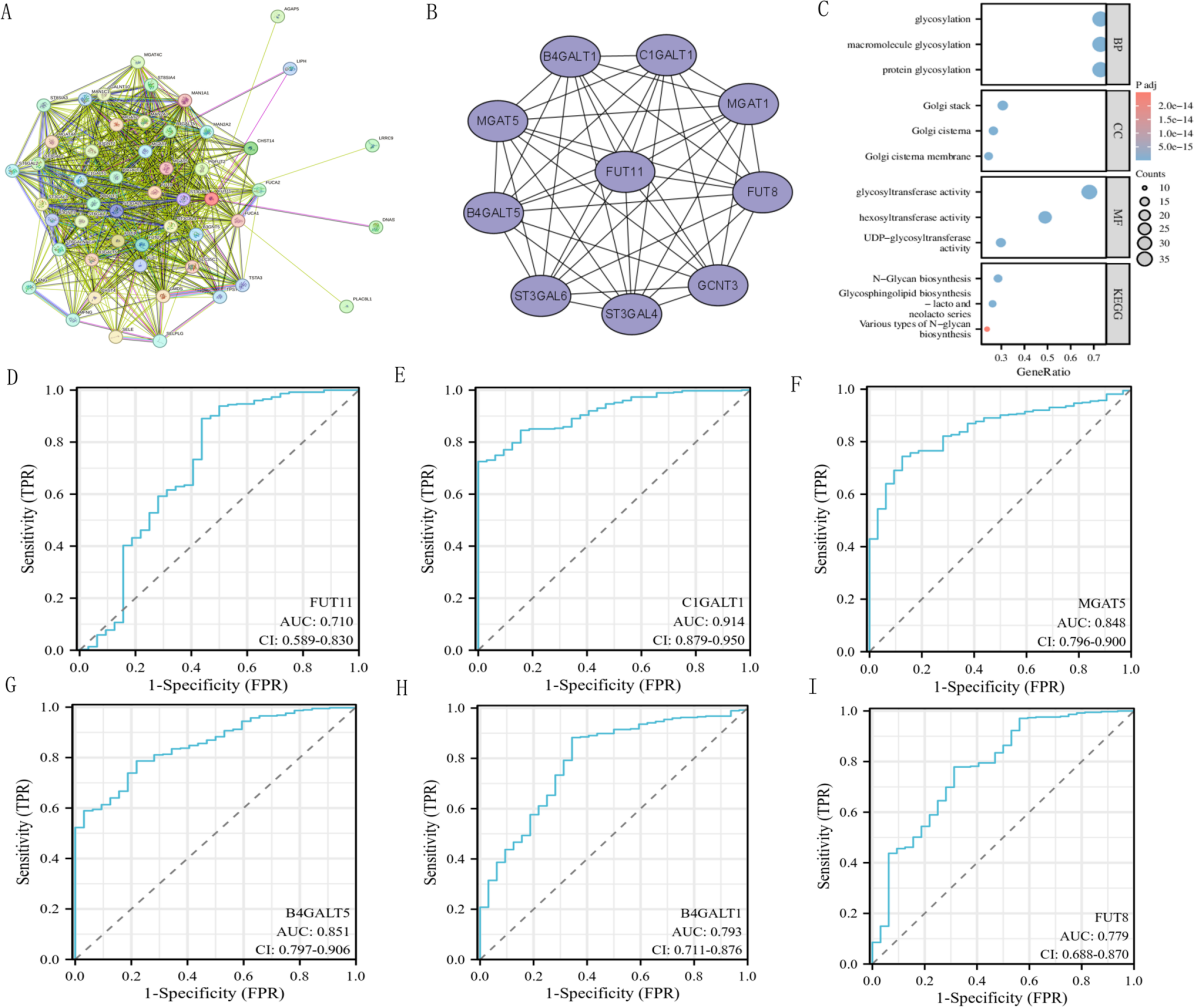
Functional enrichment of FUT11 protein interaction network and hub gene and their diagnostic ROC in GC. **A** PPI interaction network diagram of FUT11. **B** The top 10 hub genes of the PPI interaction network; **C** GO/KEGG enrichment analysis of FUT11 and hub genes; **D**–**I** Diagnostic ROC of FUT11 and hub gene

Earlier, we determined that patients with GC with high FUT11 expression had shorter OS and worse pathological staging. Based on these results, we explored which genes FUT11 interacts with and through which biological pathways it affects GC development and progression. We built a PPI network using a list of 50 genes closely related to FUT11 obtained from the STRING database, which we constructed by searching under a given threshold (Fig. 3A). We identified the first 10 hub genes in this network: ST3GAL4, ST3GAL6, MGAT1, FUT11, C1GALT1, MGAT5, B4GALT5, B4GALT1, FUT8, and GCNT3 (Fig. 3B).

Subsequently, we enriched these hub genes through GO/KEGG analysis, and the results demonstrated significant enrichment in various biological processes (BP), cell components (CC), and molecular functions (MF), all with P < 0.05 (Fig. 3C). The Key BP categories were glycosylation, macromolecular glycosylation, and protein glycosylation. The main CC categories were Golgi heap, Golgi pool, and Golgi pool membrane, while the main MF categories were glycosyltransferase activity, hexose transferase activity, and UDP glycosyltransferase activity. Additionally, the main KEGG pathways identified included *N*-glycine biosynthesis, glycosphingolipid biosynthesis lactose, new lactose series, various types of *N*-glycans, and biosynthesis. FUT11, C1GALT1, MGAT5, B4GALT1, B4GALT5, and FUT8 demonstrated good diagnostic values, with AUC values of 0.7–0.9 (Fig. 3D–I). These results indicated that FUT11 affects GC occurrence and development mainly by participating in protein glycosylation modification and has significant diagnostic value. This result is an important clue to further understand GC pathogenesis and develop new diagnostic and therapeutic strategies.

### Enrichment analysis of FUT11 co-expressed genes in GC

**Fig. 4:**
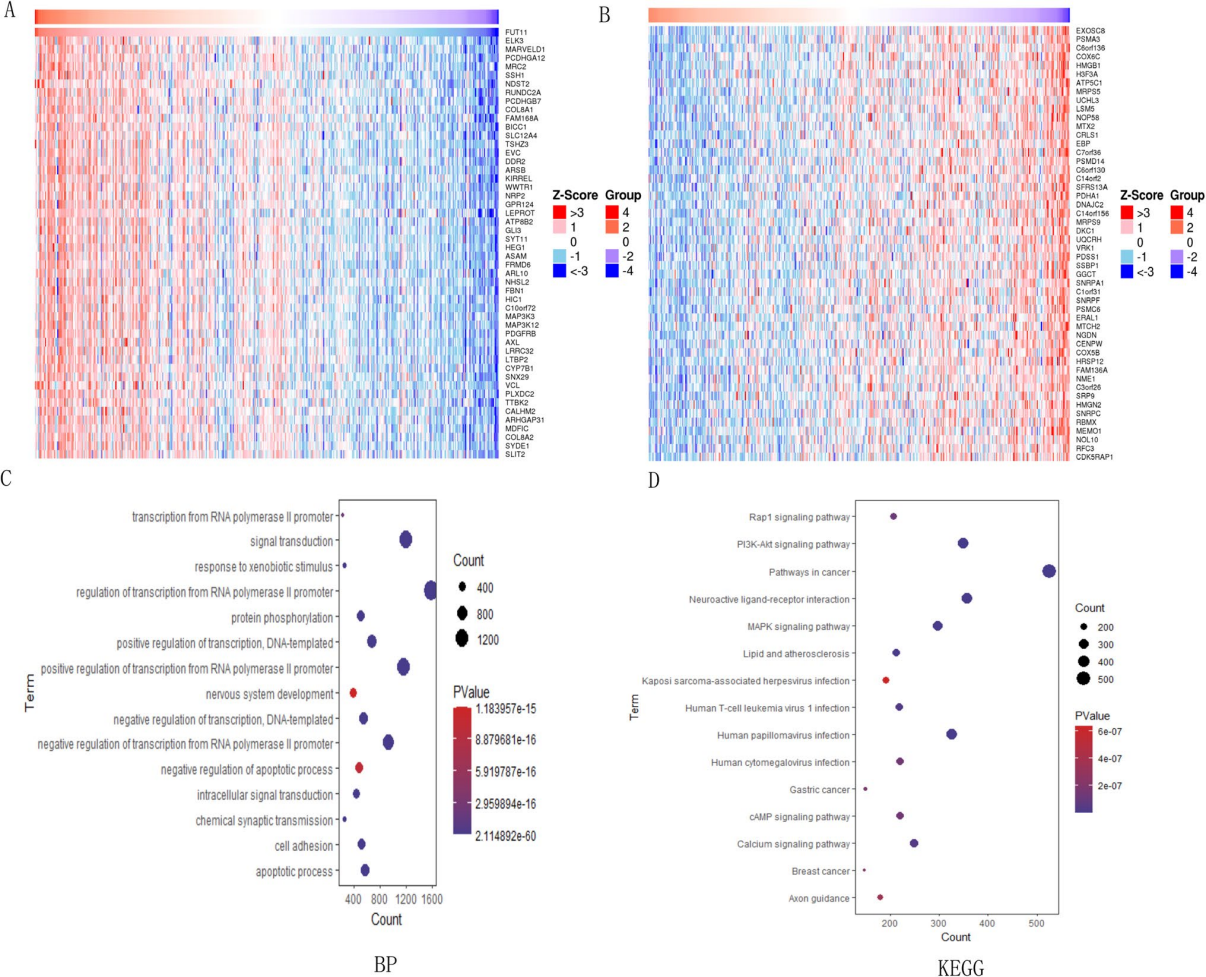
Enrichment analysis of FUT11 co expressed genes in GC. **A** The top 50 genes positively correlated with FUT11; **B** The top 50 genes negatively correlated with FUT11; **C** Biological processes related to the function of the FUT11 gene (BP); **D** KEGG enrichment analysis between co expressed genomes with FUT11 based on the Linkedomics database

The potential role and mechanism of FUT11 in GC were examined by exploring the co-expression network between FUT11 and GC using LinkedOmics. Our analysis revealed that FUT11 was positively associated with 10,801 genes and negatively associated with 9,424 genes. We screened the top 50 genes positively and negatively associated with FUT11 and visualized them using heat maps (Fig. 4A, B). We performed KEGG pathway and GO function analysis for FUT11 and its co-expressed genes (P < 0.05). The results indicated that FUT11 and its co-expressed genes were important in signal transduction, RNA polymerase II promoter transcriptional regulation, and protein phosphorylation. Additionally, these genes are crucial in cancer pathogenesis, particularly through the PI3K–AKT signaling pathway, neuroactive ligand–receptor interactions, and the MAPK signaling pathway (Fig. 4C, D). This result revealed the complex and fine mechanism of FUT11 in GC and provided clues for subsequent research and treatment strategy formulation.

### Immunoinfiltration analysis of FUT11 in GC

**Fig. 5:**
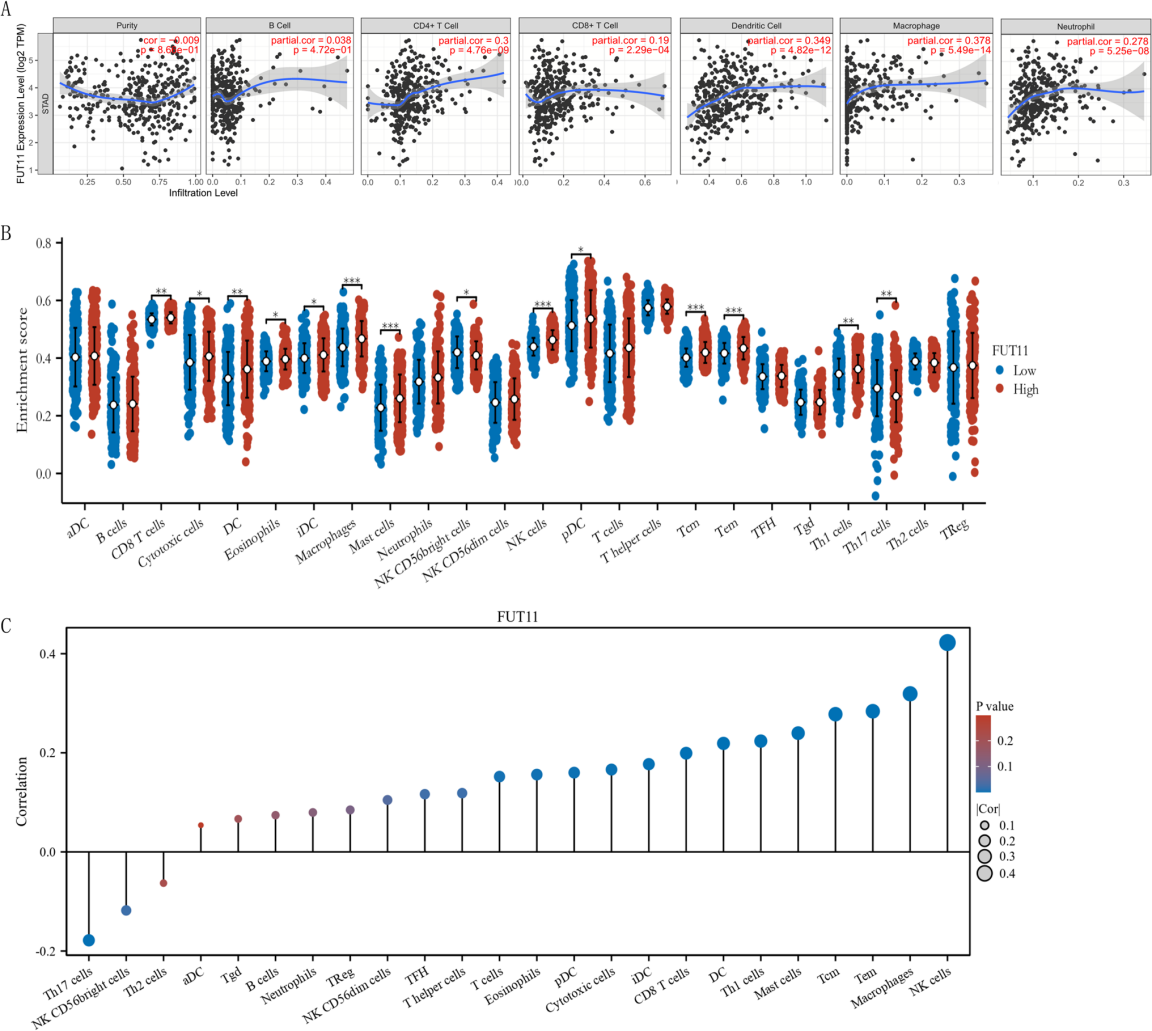
Analysis of FUT11 expression and infiltration of various immune cells. **A** Scatter plot of the correlation between FUT11 and tumor purity, B cells, CD4+ cells, CD8+ cells, dendritic cells, macrophages, and neutrophils based on TIMER2; **B** Differences in abundance between high and low expression groups of FUT11 and 24 types of immune cells; **C** The correlation between 24 types of immune cells and FUT11 expression

The critical role of immune infiltration in tumor progression has become increasingly apparent recently. Therefore, we explored the association between FUT11 expression and immune cell infiltration in GC using in-depth analysis. The TIMER2.0 public database analysis revealed a positive correlation between FUT11 expression and the degree of infiltration of CD4+ T cells, CD8+ T cells, dendritic cells, macrophages, and neutrophils in GC. This result suggested that the infiltration of these immune cells into GC tissues increases together with increased FUT11 expression. However, no correlation was observed between tumor purity and B-cell infiltration (Fig. 5A).

We also explored the intricate interactions between the immune microenvironment and FUT11 expression. We divided 375 TCGA-STAD patients with GC into high and low FUT11 expression groups according to the median FUT11 expression. The results demonstrated that the high-expression group had a higher amount of infiltration by 12 major immune cell types (CD8 T cells, cytotoxic cells, dendritic cells, eosinophils, iDC, macrophages, mast cells, NK cells, pDC, Tcm, Tem, Th1 cells) than the low-expression group (P < 0.05). Contrastingly, the high-FUT11 group had a lower amount of Th17 cells and NKCD56bright cells (Fig. 5B). This result suggested that FUT11 might suppress Th17-mediated inflammation by inhibiting Th17 cell activation and proliferation. Additionally, FUT11 might reduce the cytotoxic activity of NKCD56bright cells against tumor cells by inhibiting their killing capacity.

We detected the correlation between FUT11 mRNA expression and GC immune cell infiltration levels using ssGSEA. The results demonstrated that FUT11 expression was positively correlated with 16 immune cell types: NKCD56dim cells, TFH, T helper cells, T cells, eosinophils, pDC, cytotoxic cells, iDC, CD8 T cells, DC, Th1 cells, mast cells, Tcm, Tem, macrophages, and NK cells. Th17 and NKCD56bright cell infiltration was negatively correlated (P < 0.05) (Fig. 5C). These results suggested that high FUT11 expression might regulate eosinophil, pDC, cytotoxic cell, iDC, CD8 T cell, dendritic cell, Th1 cell, mast cell, Tcm, Tem, macrophage, and NK cell infiltration in GC tissues. Th17 and NKCD56bright cell infiltration in GC tissue was inhibited, affecting GC occurrence and development. FUT11 might attract NKCD56dim cells to the tumor site by secreting specific chemokines while simultaneously promoting TFH cell generation by regulating T helper cell activation and differentiation. Notably, Th17 cells are crucial for anti-tumor immunity, and NKCD56bright cells can directly kill tumor cells. Therefore, the negative correlation between FUT11 expression and Th17 and NKCD56 bright cells might weaken the anti-tumor immune response and reduce the immune killing capacity within the tumor microenvironment, promoting tumor growth and immune evasion.

**Fig. 6:**
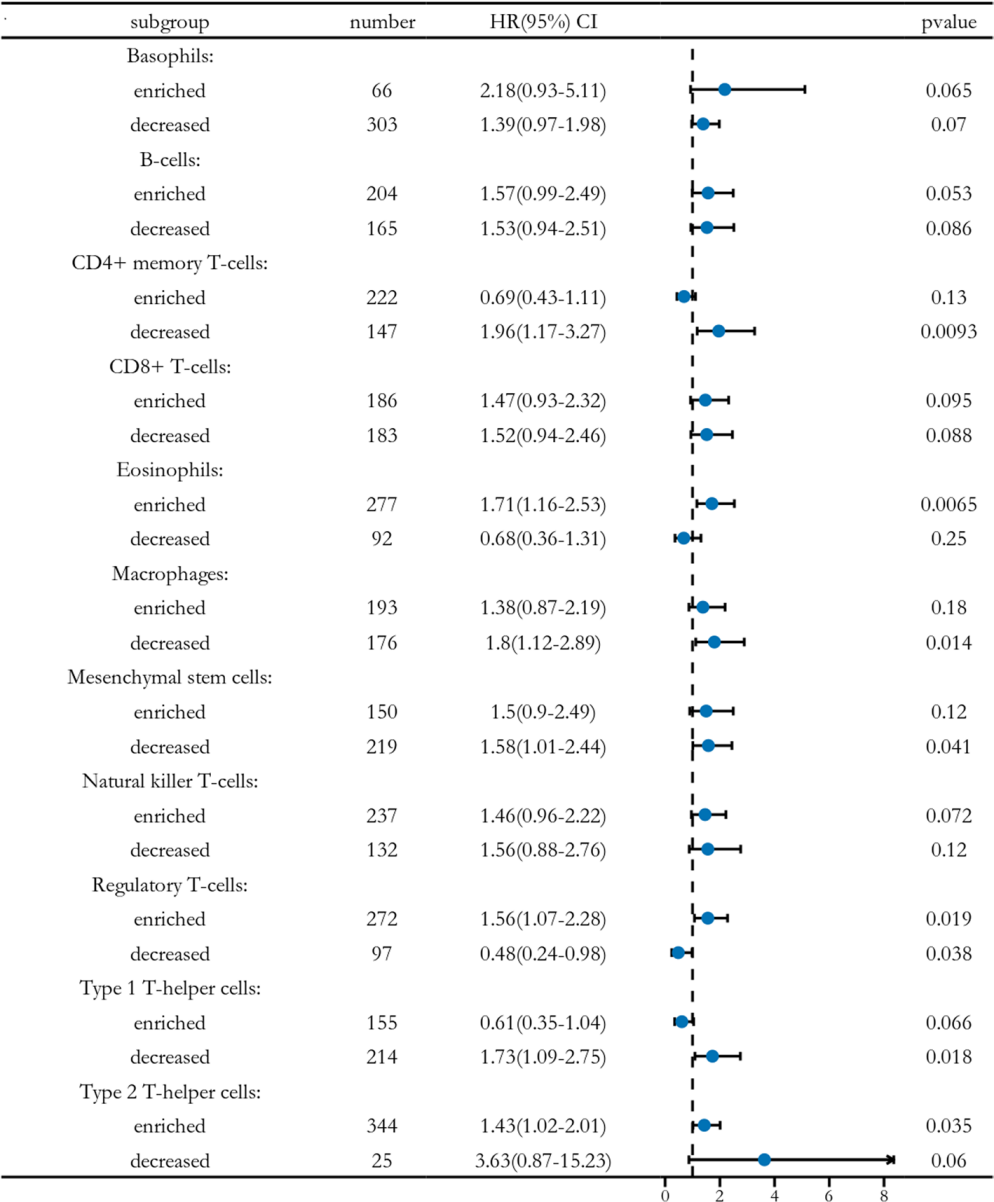
HR and p-value forest plots of FUT11 expression in KM survival analysis of gastric cancer patients under different immune cell subsets

We investigated the relationship between cancer-associated fibroblast infiltration levels and FUT11 expression across various tumor types in the TCGA dataset using TIMER2.0. Finally, we explored whether FUT11 expression might influence the prognosis of patients with GC by modulating immune cell infiltration. High FUT11 expression in the patients was associated with increased eosinophil, regulatory T cell, and type 2T helper cell infiltration. Conversely, reduced CD4+ memory T cell, macrophage, mesenchymal stem cell, and T helper type 1 cell infiltration was associated with a less favorable prognosis (Fig. 6). The results demonstrated that FUT11 is involved in the biological process of immune cells entering tumor tissues, improving the tumor microenvironment, which might affect the development of GC and patient prognosis.

### FUT11 genetic variation in GC

**Fig. 7:**
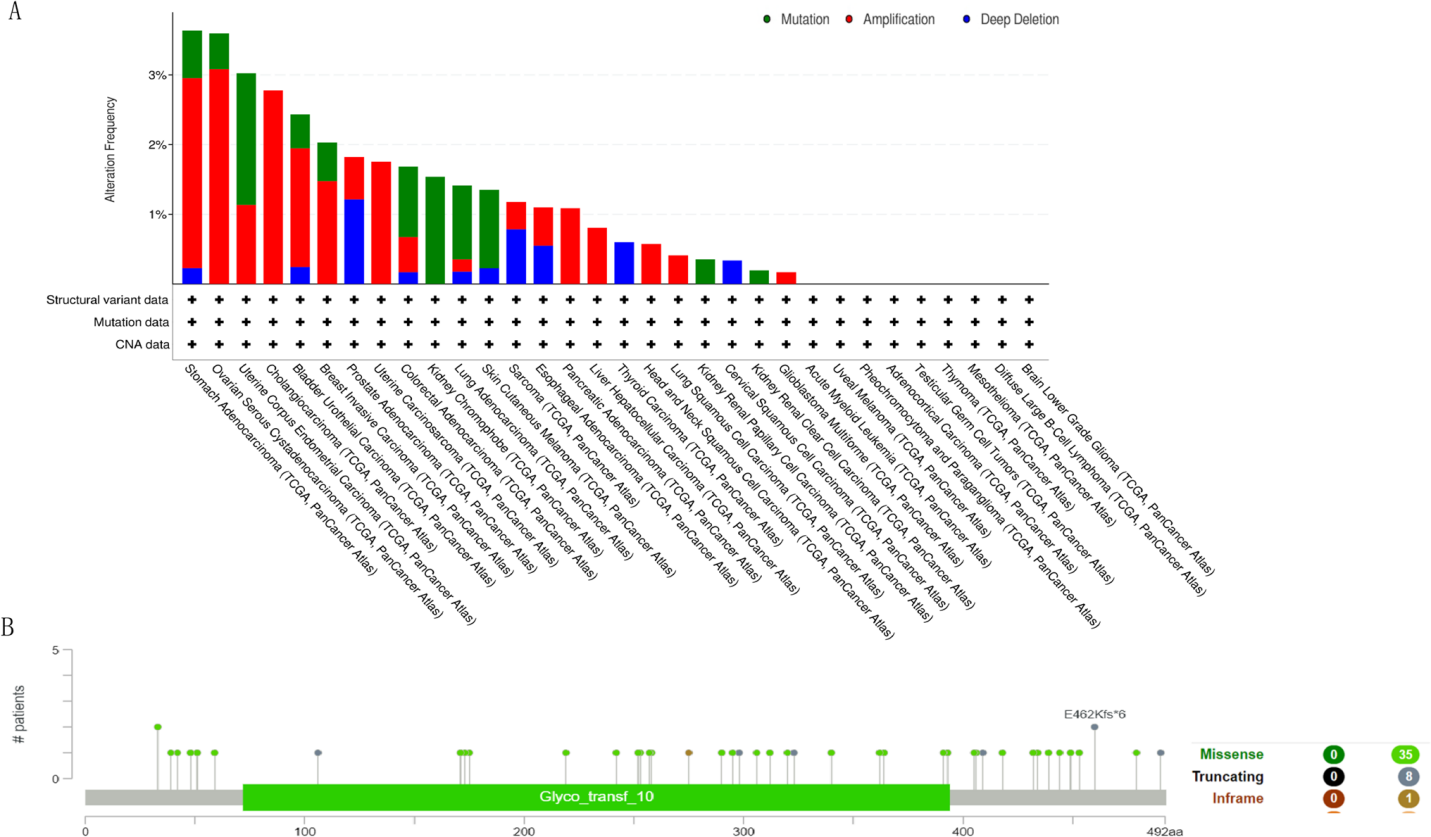
Genetic mutations in FUT11 in the cBioPortal database. **A** Histograms of FUT11 mutations in 32 types of cancer; **B** FUT11 mutation map across protein domains

FUT11 mutations in cancers were explored using the cBioPortal platform and TCGA data for mutation analysis. We covered 32 TCGA pan-cancer studies covering 10,967 samples. The analysis identified 44 mutation sites within the FUT11 gene, covering amino acids 0–492. The mutations included 35 missense mutations, eight truncated mutations, and one basal structural change, with the most common mutation site occurring at E462Kfs*6 (Fig. 7B). Missense mutations, amplifications, and deep deletions were the main mutations. Additionally, FUT11 mutations were most frequent in GC, ovarian cancer, and endometrial cancer (Fig. 7A). The pan-cancer analysis demonstrated that FUT11 was significantly amplified in GC, and missense mutations were the most dominant mutation in FUT11.

We also sought to explore the potential relationship between FUT11 gene alterations and survival and prognosis in patients with GC. However, our comprehensive analysis did not demonstrate significant effects of FUT11 gene alterations on patient outcomes. Although unexpected, these results highlighted the need for validation through clinical patient data.

### Drug sensitivity analysis of FUT11

**Fig. 8:**
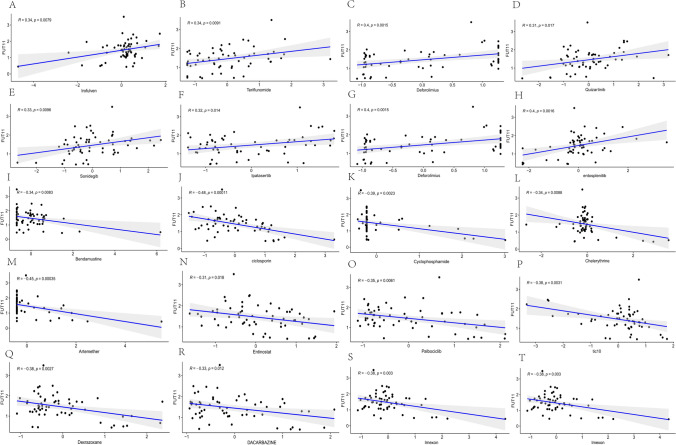
Drug sensitivity analysis of FUT11 gene expression. **A**–**T** Scatter plot of the correlation between high expression of the FUT11 gene and Irofulven, Teriflunomide, Deforolimius, Quizartinib, Sonidegib, Ipataserib, Idealisib, endosplenitib, Bendamustine, ciclosporin, Cyclophosphamide, Chelsea, Artemether, Entinostat, Palbociclib, tic10, Dexrazoxane, Dacarbazine, Ifosfamide, Imexon

Chemotherapy is one of the most important GC treatment methods, and the drug resistance of chemotherapy drugs has been the focus of research. We analyzed the intricate relationship between FUT11 gene expression and cancer treatment drugs using the CellMiner database. Our analysis revealed a striking pattern: high FUT11 expression levels were positively associated with irofulven, teriflunomide, deforolimus, quizartinib, sonidegib, ipatasertib, idelalisib, and entospletinib. This result indicated that high FUT11 expression sensitizes cells to the effects of these drugs. On the contrary, lower FUT11 expression levels were negatively correlated with bendamustine, ciclosporin, cyclophoride, chelsea, artemether, entinostat, palbociclib, TIC10, dexrazoxane, dacarbazine, ifosfamide, and imxon. This negative correlation suggested potential drug resistance when FUT11 expression is low (Fig. 8A–T). Interestingly, the FUT11 expression changes did not affect the commonly used chemotherapy drugs for GC, such as paclitaxel, oxaliplatin, tic10, cisplatin, capecitabine, and docetaxel, and there was no statistical significance. Although the FUT11 drug sensitivity analysis results in GC were unsatisfactory in gastric cancer, they revealed the potential effects of FUT11 expression on drug sensitivity and resistance.

### FUT11 expression in human GC tissue

**Fig. 9:**
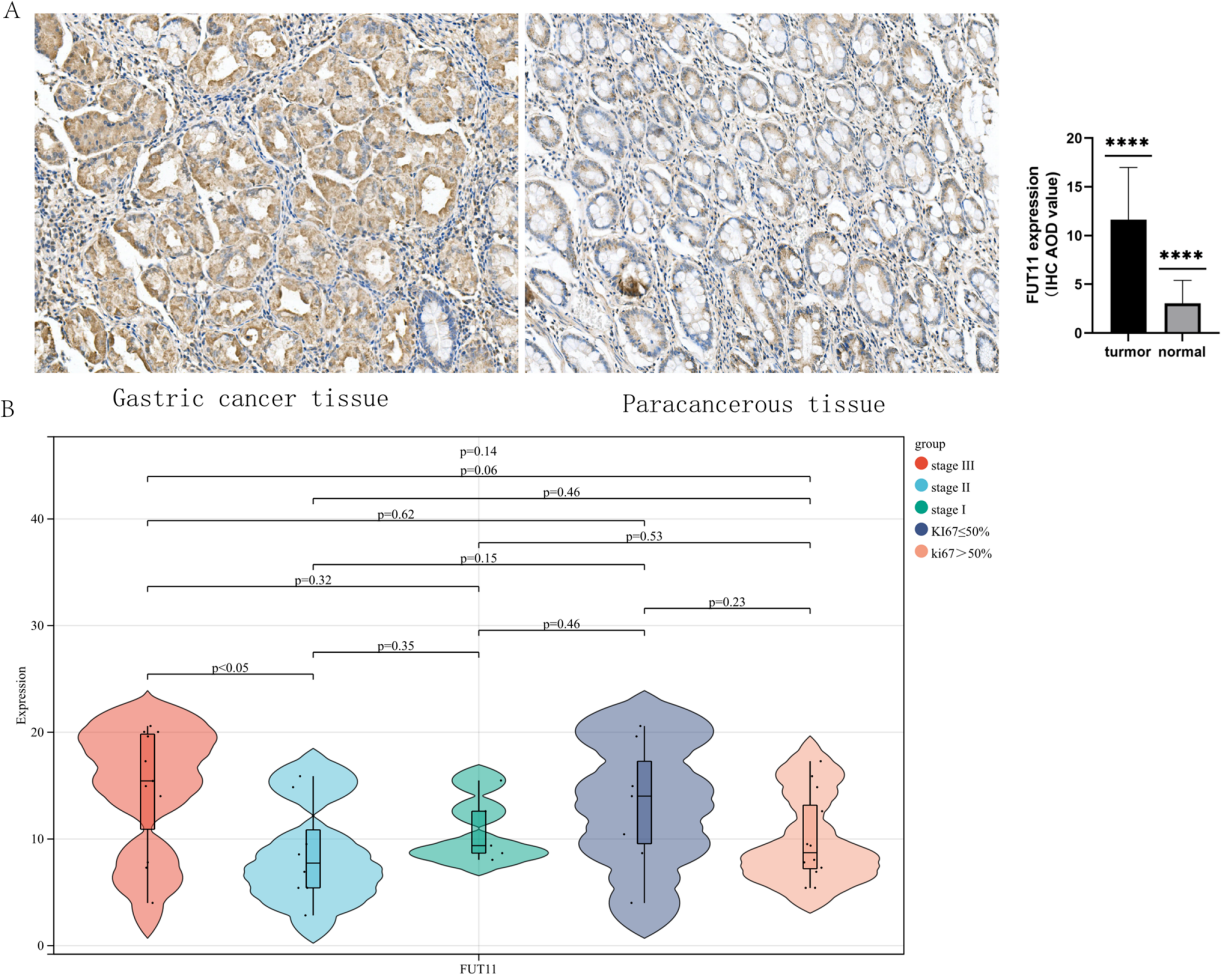
The expression of FUT11 in human gastric cancer and adjacent tissues. **A** Semi quantitative analysis (AOD value) of FUT11 in GC and adjacent tissues (IHCx200) (* * * * P < 0.0001). **B** The expression of FUT11 in gastric cancer tissues with different pathological stages and Ki-67 expression

Bioinformatics analysis results might not always be reliable. Therefore, the results were validated using in vitro experimental analysis. Immunohistochemical staining demonstrated that FUT11 was mainly located in the cytoplasm. Subsequently, we investigated FUT11 expression in 25 pairs of GC tissues and para-cancerous tissues. The immunohistochemical results demonstrated that the GC tissues had higher FUT11 expression than the para-cancerous tissues (P < 0.0001) (Fig. 9A). Further analysis of the pathological characteristics of this data set revealed that FUT11 was highly expressed in stage III GC tissues compared with stage II GC tissues (P < 0.05) (Fig. 9B), which was consistent with our earlier data analysis. However, Ki-67 expression, stage I, and stage II in GC tissue were not correlated with FUT11 expression, which might be related to the small sample size.

## Discussion

GC is a prevalent malignant tumor in the digestive tract, marked by a complex tumor microenvironment. The latest World Health Organization report indicates that around 1 million new cases of GC are diagnosed annually [1]. Due to its subtle onset and swift progression, most patients are diagnosed at an advanced stage. Even with radical gastrectomy and adjuvant chemotherapy, the 5-year survival rate for patients with stage II and beyond drops significantly [27]. Traditional treatments, including radical gastrectomy, chemotherapy, intraperitoneal perfusion chemotherapy, and more recent targeted immunotherapies, have been utilized for GC patients. However, the prognosis has not markedly improved due to the toxicity of chemotherapy drugs, challenges in identifying suitable candidates for targeted therapies, and increasing drug resistance [28].

Given these challenges, there is an urgent need to investigate the molecular mechanisms by which GC occurs and develops. Fucosylation modification is one of the main parts of glycosylation modification after protein transcription and translation. Abnormal glycosylation is a defining feature of tumor development, directly contributing to tumor progression and metastasis [4], making it a promising target for cancer therapy. As a fucosylated transferase, FUT11 is involved in the occurrence and development of various tumors, and the biological behavior of various cancers.

In the present study, we conducted a public database analysis and determined that FUT11 expression was significantly increased in GC tissues compared to neighboring tissues, which was confirmed by immunohistochemical staining. Furthermore, FUT11 demonstrated diagnostic potential in distinguishing GC tissues from neighboring tissues. We conducted survival prognosis and univariate and multivariate Cox analysis using TCGA-STAD data to understand the survival prognosis and related clinical significance of abnormal FUT11 expression in GC, and the results demonstrated that high FUT11 expression in GC was associated with poor survival prognosis. The ROC curve analysis confirmed the clinical diagnostic value of FUT11 in the patients. The results also highlighted that FUT11 expression was more pronounced in patients with advanced GC, indicating its potential role in disease progression. Additionally, highly expressed FUT11 was more strongly correlated with the N stage, suggesting that FUT11 expression might affect the prognosis of patients with GC by affecting lymph node metastasis.

The drug sensitivity analysis revealed that high FUT11 expression demonstrated high sensitivity for some cancer treatment drugs. These results suggested that FUT11 might be involved in GC progression and presents a potential direction for cancer therapy. We constructed a FUT11 protein interaction network and conducted GO and KEGG enrichment analysis to gain a deeper understanding of the genes related to GC occurrence and development of GC. Finally, we correlated the FUT11 expression level with the level of cancer immune cell infiltration, and determined that FUT11 is involved in the biological process of immune cells entering tumor tissues, improving the tumor microenvironment, which might affect the development of GC and patient prognosis. Therefore, FUT11 has great potential as an immune-related biomarker in GC. Our study provides a reference for the mechanism by which FUT11 participates in GC development.

Tumor microenvironments, including immune cells, tumor cells, fibroblasts, and other cell clusters, have been extensively studied in recent years and profoundly affect cancer progression. Tumor cells can stimulate an immune response, which is suppressed in some patients to create the right occasion for tumor cell growth, metastasis, chemotherapy resistance, and immune tolerance [29]. FUT11 might affect the GC immune microenvironment [30], suggesting that FUT11 might be involved in the immune response. Therefore, how FUT11 participates in the tumor microenvironment and affects tumor-infiltrating immune cells in GC should be investigated. The TIMER2.0 database demonstrated that FUT11 affected tumor-infiltrating immune cells in GC. FUT11 expression was positively correlated with CD4+ T cell, CD8+ T cell, dendritic cell, macrophage, neutrophil, and cancer-associated fibroblast infiltration. ssGSEA confirmed that FUT11 expression was positively correlated with NK CD56dim cells, TFH, T helper cell, T cell, eosinophil, pDC, cytotoxic cell, iDC, CD8 T cell, DC, Th1 cell, Mast cell, Tcm, Tem, macrophage, and NK cell infiltration, while it was negatively correlated with Th17 and NK CD56bright cell infiltration. FUT11 might promote tumor growth and immune evasion by attracting NKCD56dim cells to the tumor site by secreting specific chemokines. Furthermore, FUT11 can facilitate TFH cell generation by modulating T helper cell activation and differentiation. Th17 cells are differentiated from CD4(+) T cells. Notably, Th17 cells are crucial for anti-tumor immunity, and NKCD56bright cells can directly kill tumor cells. Therefore, the negative correlation between FUT11 expression and Th17 and NKCD56bright cells might weaken the anti-tumor immune response and reduce the immune killing capacity within the tumor microenvironment. The increased NK and CD8+ T cells can enhance the anti-tumor immune response by secreting cytokines and releasing perforin and granzymes [31]. DC are the most powerful antigen-presenting cell, and their infiltration can promote the presentation of tumor-associated or tumor-specific antigens to T cells, triggering a primary immune response [32]. In the present study, FUT11 positively regulated the infiltration of T cells, DC, CD8 T cells, and other cells to promote anti-tumor activity. Therefore, FUT11 is involved in anti-tumor activity and tumor microenvironment regulation in GC by participating in cellular immunity.

Immune checkpoint inhibition is a promising method for treating advanced gastric cancer, driving clinical research and practice forward [32, 33]. Tumor cells evade the host’s immune response through various mechanisms [34]. Multiple immune checkpoint inhibitors target different molecular pathways on tumor and immune cells, highlighting the importance of co-inhibitory signaling pathways such as CTLA-4 and PD-1/PD-L1 in tumor-induced immunosuppression. For example, PD-1 expressed on T cells, B cells, and bone marrow cells binds to PD-L1 on antigen-presenting cells and tumor cells, activating immunosuppressive pathways that inhibit T cell function and promote tumor immune escape [35]. PD-1/PD-L1 inhibitors show great promise in treating advanced cancers [36]. Conversely, CTLA-4 binds to B7 on antigen-presenting cells, blocking its interaction with the CD28 receptor on CD4 T cells, thus depriving T cells of their co-stimulator signal [37], while anti-CTLA-4 antibodies release T cells from this inhibition. Blocking therapy has been explored in HER2-positive GC, including pembrolizumab, nivolumab, and ipilimumab, in standard first-line therapy along with trastuzumab [38]. Numerous clinical investigations have reported that only a small percentage of patients receive effective immunotherapy, while the vast majority of patients receive unsatisfactory immunotherapy. Predicting the efficacy of various immunotherapies at different GC stages remains a challenge, which requires continued exploration of potential biomarkers. These include PD-L1 expression, tumor mutation load (TMB), MSI/MMR status, EBV infection, circulating tumor DNA (ctDNA), and gut microbiota. Reliable clinical trials and targeted immunotherapies potentially greatly improve the survival rates of patients with GC. In the present study, immune cell infiltration analysis revealed that FUT11 is involved in the biological process of immune cells entering tumor tissues, improving the patient’s tumor microenvironment, which might affect the development of GC and patient prognosis. This result suggested that FUT11 is a tumor prognosis predictor and is also a possible tumor therapy target, providing a novel molecule with immunomodulatory function for GC treatment.

We constructed a FUT11 PPI network and screened for related hub genes. We also studied the role of the genes co-expressed with FUT11 in GC with LinkedOmics to explore the potential function of FUT11 in GC. We determined that FUT11 is involved in multiple signaling pathways in tumor cells, e.g., the PI3K–AKT signaling pathway, neuroactive ligand–receptor interactions, and the MAPK signaling pathway. The PI3K–AKT signaling pathway is an important signaling pathway involved in normal cellular processes. The pathway has a wide range of roles in different diseases and cancer progression. Its abnormal activation regulates autophagy, epithelial–mesenchymal transition, apoptosis, chemotherapy resistance, and metastasis in many cancers [39].

Hypoxia is a common marker of tumor cell microenvironment growth. The imbalanced oxygen supply and tumor cell consumption can trigger hypoxia reactions between numerous molecules and tumor cells; alter tumor cell metabolism; promote tumor cell survival, invasion, and migration; generate new blood vessels; and enhance the resistance to ionizing radiation and various chemotherapies to help tumor cells in adapting rapidly to hypoxia environments [40]. Hypoxia-inducer factor 1 (HIF-1) is a key downstream protein of the PI3K–AKT signaling pathway, which is closely related to the oxygen concentration in the environment. Maintaining stable HIF-1 protein levels under normal oxygen conditions is essential, and HIF-1 levels increase rapidly under hypoxia conditions. HIF-1α is involved in acute hypoxia associated with erythropoietin, while HIF-2α is associated with chronic hypoxia [41]. Ruan et al. reported that HIF-1α binds to the FUT11 promoter and increases its transcription and co-expression with FUT11 in hepatocellular carcinoma tissues, suggesting that FUT11 may be the target gene of HIF-1α and potentially accelerates hepatocellular carcinoma cell proliferation and migration. Accordingly, FUT11 might be a powerful target for blocking the stimulative effect of hypoxia on hepatocellular carcinoma [13]. Cao et al. reported that downregulating FUT11 inhibited GC progression through the PI3K–AKT pathway [42]. Based on our previous series of bioinformatic analyses on the relationship between FUT11 and gastric cancer, we speculate that FUT11 may bind the FUT11 promoter through HIF-1α, increase its transcription and co-expression with FUT11, and participate in the PI3K–AKT signaling pathway to regulate GC occurrence and development. Nevertheless, this idea should be confirmed with more basic experiments.

## Conclusions

We determined that high FUT11 expression in GC is associated with a poorer prognosis. FUT11 is involved in anti-tumor activity and tumor microenvironment regulation in GC by participating in cellular immunity. While the results enhance our understanding of the relationship between FUT11 and GC, it has some limitations. First, our results relied on public data sources, which limited the applicability of the results under specific conditions. Second, the mechanism of action of FUT11 and its effect on GC clinical outcomes should be verified using molecular studies. Additionally, the key FUT11-related signaling pathways must be verified, such as how FUT11 regulates GC occurrence and development through the PI3K–AKT signaling pathway. Finally, the mechanism of FUT11 regulation of immune cell infiltration requires further examination. Finally, we emphasize again that our findings have important implications for GC treatment and prognosis, but more research is needed to confirm our findings and translate them into solid clinical value before they can be applied to clinical practice to better benefit the patient population.

### Supplementary Information

Supplementary Material 1.

## Data Availability

The standardized datasets involved in this study were sourced from the TCGA (https://portal.gdc.cancer.gov/) and GEO (https://www.ncbi.nlm.nih.gov/geo/) databases, and all data were from different biological samples, without considering repeated testing.The data and materials that support the findings of this study are available on request from Discover Oncology.

## Declarations

### Publication statement

This article was previously published in preprint, author and name: “Yanqing Huang, Xiaoying Yang, Mengda Wei et al. FUT11 Expression and Its Clinical Significance in Gastric Cancer. Research square. November 2023. 10.21203/rs.3.rs-3565964/v1”.
