# Supplementary figures and images for: FUT11 expression in gastric cancer: its prognostic significance and role in immune regulation
Source: Discov Oncol. 2024 Jun 28;15:250. doi: 10.1007/s12672-024-01120-y (PMC11213843; doi:10.1007/s12672-024-01120-y)

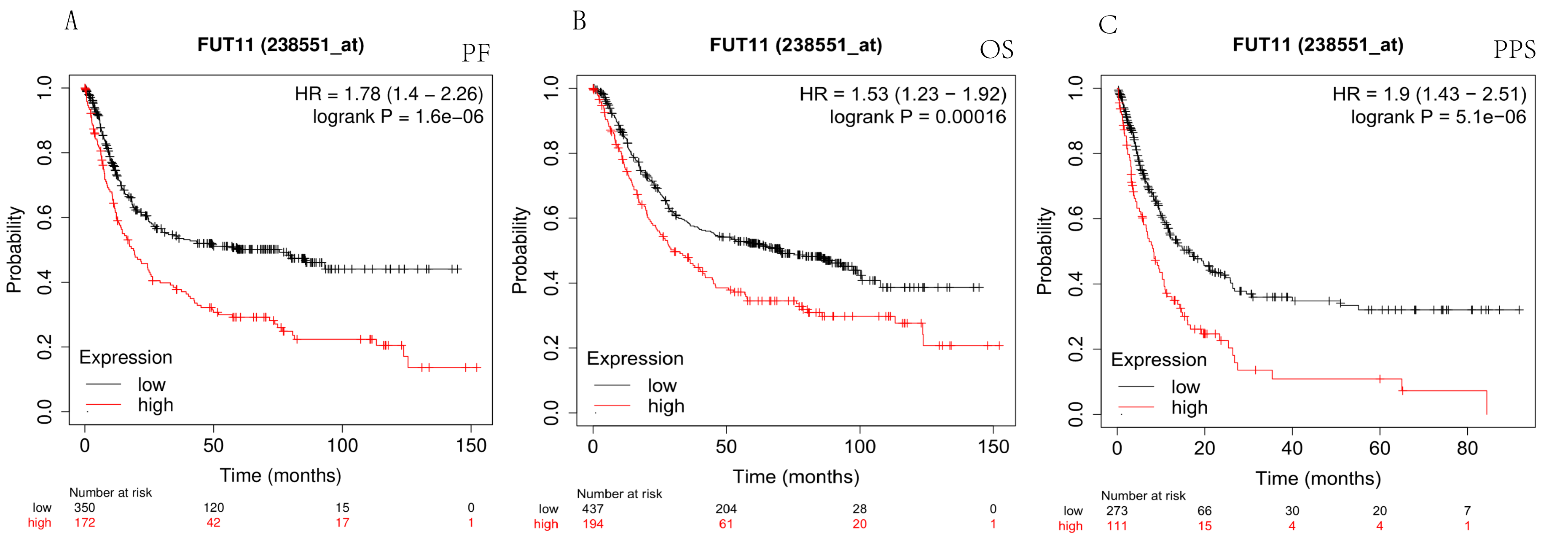

Supplement: Supplementary file 1 — Supplementary Material 1. [file 12672_2024_1120_MOESM1_ESM.tiff]
